# High-throughput RNA sequencing reveals the effects of 2,2′,4,4′ -tetrabromodiphenyl ether on retina and bone development of zebrafish larvae

**DOI:** 10.1186/s12864-014-1194-5

**Published:** 2015-01-23

**Authors:** Ting Xu, Jing Zhao, Daqiang Yin, Qingshun Zhao, Bingzhi Dong

**Affiliations:** Post-doctoral Research Station of Civil Engineering, Tongji University, Shanghai, 200092 China; Key Laboratory of Yangtze River Water Environment, Ministry of Education, College of Environmental Science and Technology, Tongji University, Shanghai, 200092 China; Model Animal Research Center, MOE Key Laboratory of Model Animal for Disease Study, Nanjing University, Nanjing, 210061 China

**Keywords:** Polybrominated diphenyl ethers, RNA sequencing, Zebrafish larvae, Functional enrichment analysis, Visual perception

## Abstract

**Background:**

2,2′,4,4′-Tetrabromodiphenyl ether (BDE47) is a prevalent environmental pollutant and has been demonstrated to be a serious toxicant in both humans and animals, but little is known about the molecular mechanism underlying its toxic effect on the early development of vertebrates. BDE47-treated zebrafish larvae were found to present the light-related locomotion reduction in our previous study, therefore, we aimed to use high throughput sequencing to investigate the possible reasons from a transcriptomic perspective.

**Results:**

By exposing zebrafish embryos/larvae to 5 μg/l and 500 μg/l BDE47, we measured the influence of BDE47 on the mRNA expression profiles of zebrafish larvae until 6 days post-fertilization, using Illumina HiSeq 2000 sequencing. Differential expression analysis and gene enrichment analysis respectively revealed that a great number of genes, and gene sets based on two popular terminologies, were affected by the treatment of 500 μg/l BDE47. Among them, BDE47 caused changes in the retinal metabolism and related biological processes involving eye morphogenesis and visual perception, as confirmed by disordered photoreceptor arrangement and thickened bipolar cell layer of larval retina from histological observations. Other altered genes such as *pth1a* and collaborative cathepsin family exhibited disrupted bone development, which was consistent with the body curvature phenotype. The transcriptome of larvae was not significantly affected by the treatment of 5 μg/l BDE47, as well as the treatment of DMSO vehicle.

**Conclusions:**

Our results suggest that high BDE47 concentrations disrupt the eye and bone development of zebrafish larvae based on both transcriptomic and morphological evidences. The abnormal visual perception may result in the alteration of dark adaption, which was probably responsible for the abnormal larval locomotion. Body curvature arose from enhanced bone resorption because of the intensive up-regulation of related genes. We also proposed the larval retina as a novel potential target tissue for BDE47 exposure.

**Electronic supplementary material:**

The online version of this article (doi:10.1186/s12864-014-1194-5) contains supplementary material, which is available to authorized users.

## Background

Polybrominated diphenyl ethers (PBDEs) are commonly used as flame retardants. Although the use of commercial mixtures such as pentabromodiphenyl ether and octabromodiphenyl ether in products has been banned or limited by the European Union, the U.S., and other countries, PBDE residues in the environment and in animal tissues still pose a serious threat [1,2]. Among PBDE congeners, 2,2′,4,4′-tetrabromodiphenyl ether (CAS: 5436-43-1) is the most frequently detected one [3], and previous studies have demonstrated it to be the most toxic [4,5]. Exposure to BDE47, an endocrine-disrupting chemical, can cause multiple adverse effects, and current research has focused on the examination of its endocrine-disrupting activity and neurobehavioral toxicity [3,6]. The best-known mechanism of BDE47 toxicity involves its ability to impair the homeostasis and function of thyroid hormone (TH) in animals because of its structural similarity to THs such as triiodothyronine (T3) and thyroxine (T4). Moreover, BDE47 can lead to various types of developmental neurotoxicity, including long-lasting behavioral alterations, by interfering with signal transduction pathways and by damaging neuron cell viability [7].

Recently, deep sequencing (next-generation RNA sequencing) has become a fast-growing high-throughput technology. Compared to microarrays, deep sequencing is independent of predefined probe sets, thereby permitting the detection of novel transcripts and alternative splicing [8]. However, thus far, while RNA sequencing has been increasingly used in toxicological studies of rodents and humans, it has only been employed for toxicological studies of aquatic species in a few studies [9,10]. In our previous study, 500 μg/L of BDE47 caused a significant reduction of larval locomotion specifically at the switch between light and dark periods; the effects seemed to be related to light stimulus [11]. The evidence for this was that a PBDE mixture, DE-71, containing BDE47 was found to cause biochemical changes in the larval eye as measured by optokinetic responses and phototaxis tests [12]. Therefore, to validate our hypothesis and understand the novel toxic mechanism of BDE47, we employed deep sequencing to obtain and analyze whole-transcriptome information for zebrafish larvae after exposure to BDE47.

In this study, the Illumina HiSeq sequencing platform was utilized to investigate the influence of PBDEs on the gene expression profiles of zebrafish larvae. We chose 6 day post-fertilization (dpf) zebrafish larvae to keep aligned with our previous work [11], and zebrafish at this stage already have many well-developed organs, for example retina. First, this study discovered that in 6 dpf zebrafish, BDE47 exposure led to major adverse effects, including disrupting the visual perception and bone development of larvae. Furthermore, the reduction of zebrafish larval locomotion was probably caused by the disruption of retinol metabolism by light stimulus, and body curvature was probably caused by abnormal bone development induced by BDE47.

## Results

### Characterization of the transcriptome of zebrafish larvae

The original RNA-Seq data are available at the Gene Expression Omnibus database (accession number GSE59968). Sequencing produced approximately 48 M total reads from a blank control sample, 56 M total reads from a sample of 0.1% DMSO vehicle, 42 M total reads from a sample of 5 μg/L BDE47, and 38 M total reads from sample of 500 μg/L BDE47. Of the total number of reads in these four groups, the mapping/unique mapping rates were 89.05%/86.67%, 89.42%/87.11%, 90.21%/88.06%, and 89.15%/86.87%, respectively. Although the sequencing depth in this study was lower than that in some previous experiments [13], high unique mapping rates provided the basis for adequate analysis. 21098 transcripts were detected in at least one of four groups. Excluding 753 non-coding RNA and 521 miscellaneous RNA, here we used 19824 mRNA transcripts in further steps.

Because TMM normalization was considered to be superior to reads per kilobase of the transcript per million mapped (RPKM) normalization in Illumina-based RNA sequencing [14], TMM was adopted as our normalization strategy. The most abundant transcripts in the four groups, including *si*, *myhz1*, *ef1a*, *LOC100535672*, *try*, *pvalb2*, *myhz2*, *krt4*, *hsp90ab1*, and *pvalb1*, varied slightly in content across all treatments. However, the 500 μg/l BDE47-treated group had more distinct impacts on the abundance of transcripts than the other 3 groups (Additional file 1: Table S1).

### Differential expression of genes with BDE47 treatments

**Figure 1:**
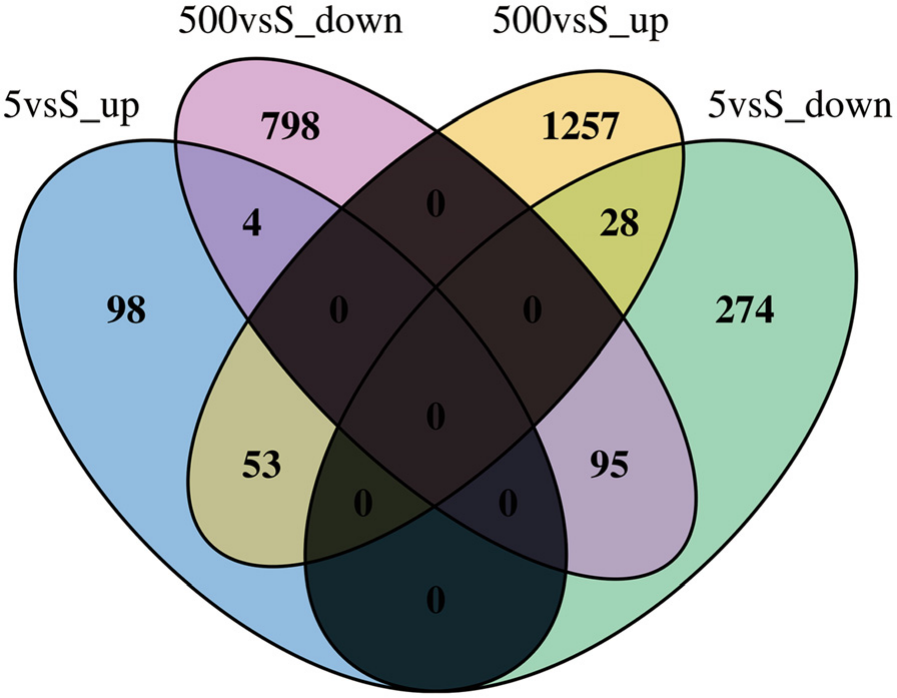
**Venn diagram showing the effects of BDE47 exposure on the detected genes.** Genes were grouped on the basis of their expression changes under different BDE47 concentrations. Expressions were evaluated on the basis of the number of reads per gene model. The shaded areas represent the existence of no intersection between two subsets.

The differentially expressed genes of zebrafish larvae treated with two BDE47 concentrations are shown as a Venn diagram (Figure 1). By comparing BDE47 treatments with the DMSO solvent control, 2235 transcripts were affected after 500 μg/L exposure, of which 1338 were up-regulated and 897 were down-regulated, and 552 transcripts were affected after 5 μg/L exposure, of which 155 were up-regulated and 397 were down-regulated. These values clearly indicate that exposure to a high BDE47 concentration resulted in a higher number of up-regulated genes. The intersections of the Venn diagram further revealed that less than 4% of up-regulated genes in the 500 μg/L BDE47-treated group were also up-regulated in the 5 μg/L BDE47-treated group, and the expression trends of a total of 32 genes reversed with increasing BDE47 concentration. These features suggested that exposure to different BDE47 concentrations resulted in different types of biological effects in the zebrafish development process. The maximum FC of genes was 15.67 (zgc:194355) when exposed to 5 μg/L BDE47 and up to 115.04 (hsp70.2, LOC100535101) when exposed to 500 μg/L BDE47. Complete lists of differently expressed genes treated with both BDE47 concentrations are shown in Additional files 2 and 3: Tables S2 and S3.

The most significant genes (FC > 10 or FC < 0.1) of zebrafish larvae after exposure to 500 μg/L BDE47 are shown in Table 1. Excluding those genes that were totally inhibited in the exposure group and with a TMM value < 1 in the DMSO group, 49 up-regulated and 22 down-regulated genes were present. Of the four most dramatically up-regulated genes, three transcripts encoded heat shock cognate (HSC) proteins: their mean FC was up to 100.59. Unlike canonical heat-shock proteins, the HSC proteins constitutively expressed and functioned during normal cellular processes; however, their up-regulation still reflected immune stress such as inflammation, infection, and cancer [15]. In the range of 10 < FC < 50, the notable transcripts were those encoding cathepsin and PIM1 enzymes. The protein products of cathepsin-related transcripts were similar to those of human cathepsin V, a protease highly homologous to mouse cathepsin L [16]. The Pim1 oncogene is involved in multiple human cancers, and enhanced Pim1 expression would benefit vision formation of zebrafish [17].

**Table 1:** **Most significant genes changed their expressions in zebrafish exposed to 500 μg/l BDE47 (FC > 10 or FC < 0.1)**

**Gene symbol**	**Accession ID**	**BLAST**	**Fold change**	**Style**	**Counts**_**500**	**Counts**_**S**
*hsp70.2*	XP_003198158	HSPA8	115.04	up	23095.632	200.760
*pth1a*	NM_212950		112.61	up	356.029	3.162
*hsp70*	NM_131397	HSPA8	111.46	up	21407.932	192.066
*hsp70l*	NM_001113589	HSPA8	75.27	up	47595.391	632.316
*LOC100536983*	XP_003200216		56.47	up	3123.057	55.328
*fosl1a*	NM_001161552	FOSL2	53.77	up	1997.507	37.149
*Fosb*	NM_001007312	FOSB	51.63	up	367.272	7.114
*lepb*	NM_001030186		49.47	up	391.007	7.904
*MGC174857*	NM_001103115	CTSV	37.93	up	59.963	1.581
*hspb9*	NM_001114705		27.69	up	7746.431	279.800
*il12a*	NM_001007107		26.87	up	42.474	1.581
*mmp13a*	NM_201503	MMP13	26.62	up	3219.247	120.930
*si:ch211-199o1.2*	NM_001100062	HSPA1L	24.38	up	1618.993	66.393
*lepa*	NM_001128576		23.71	up	112.430	4.742
*LOC557301*	XP_685429	CARTPT	21.11	up	5723.939	271.105
*LOC100538045*	XP_003197928		20.55	up	32.480	1.581
*LOC570404*	XP_698974		20.39	up	161.150	7.904
*MGC174155*	NM_001103118	CTSV	18.97	up	59.963	3.162
*si:dkey-26 g8.4*	XP_003199634	CTSV	18.97	up	59.963	3.162
*LOC561147*	XP_689643	PIM1	18.97	up	44.972	2.371
*MGC174152*	NM_001105680	CTSV	17.12	up	81.199	4.742
*atf3*	NM_200964	ATF3	16.79	up	7218.010	429.975
*LOC100333562*	XP_002666978	HIST1H4F	16.60	up	26.234	1.581
*LOC100003896*	NM_001115069		16.20	up	51.218	3.162
*LOC100000332*	XP_002662857	RNF182	15.21	up	4148.669	272.686
*si:ch211-231 m23.4*	XP_002665089	FFAR3	15.01	up	23.735	1.581
*il11b*	XP_003200597		15.01	up	23.735	1.581
*si:dkey-239j18.3*	XP_001341714	CTSV	15.01	up	23.735	1.581
*LOC100332718*	XP_002666833	GDPGP1	14.75	up	34.978	2.371
*timp2b*	NM_213296	TIMP2	14.53	up	3892.578	267.944
*LOC100538016*	XP_003200235	BTG3	14.07	up	1000.628	71.136
*LOC797181*	XP_001337638	CXCR1	13.83	up	43.723	3.162
*LOC561346*	XP_689846	PIM1	13.75	up	108.682	7.904
*LOC100007777*	XP_001346148	PIM1	12.12	up	86.196	7.114
*LOC100334610*	XP_002663270		12.05	up	228.608	18.969
*ifnphi1*	NM_207640		11.85	up	18.738	1.581
*ptrh1*	NM_001013330	PTRH1	11.78	up	595.879	50.585
*mmp9*	NM_213123	MMP9	11.61	up	5094.331	438.669
*insb*	NM_001039064	INS	11.59	up	54.966	4.742
*sgk2b*	NM_001076564	SGK1	11.30	up	938.166	82.991
*si:dkey-85 k15.6*	XP_001344298		11.06	up	34.978	3.162
*LOC100537455*	XP_003199799	ADTRP	11.06	up	26.234	2.371
*LOC795066*	XP_001335163	CASP10	10.75	up	42.474	3.952
*LOC100007785*	XP_001920525	PIM1	10.59	up	83.698	7.904
*il1b*	NM_212844		10.46	up	760.777	72.716
*ctsl1b*	NM_131198	CTSV	10.27	up	16.240	1.581
*tcap*	XP_684103	TCAP	10.22	up	4128.682	403.892
*LOC561283*	XP_001920442	PIM1	10.16	up	112.430	11.066
*cyp2k6*	NM_200509	CYP2C19	10.15	up	738.291	72.716
*LOC100535212*	XP_003199890	NXF1	0.099	down	1.249	12.646
*LOC100537950*	XP_003200691		0.099	down	1.249	12.646
*LOC100151419*	XP_001922180	IRGC	0.093	down	1.249	13.437
*LOC794295*	XP_001334228	CBLN4	0.088	down	7.495	85.363
*LOC100536146*	XP_003198781	TBX5	0.053	down	1.249	23.712
*LOC100537396*	XP_003198045	GIMAP7	0.049	down	1.249	25.293
*LOC100538301*	XP_003200448	TTLL5	0.045	down	1.249	27.664
*cyp2aa8*	NM_001006080	CYP2J2	0.040	down	2.498	63.232
*si:dkey-58f10.12*	XP_002662081		0.026	down	1.249	47.424
*aqp8b*	NM_001114910	AQP8	0	down	0	20.550
*dicp1.1*	LOC100007383		0	down	0	19.760
*or115-11*	NM_001130806	OR7C1	0	down	0	18.969
*ora3*	XP_002666147		0	down	0	16.598
*LOC100535486*	XP_003198138	AQP8	0	down	0	14.227
*LOC100535815*	XP_003198338	ZFP2	0	down	0	14.227
*LOC567550*	XP_005162589	G2E3	0	down	0	13.437
*aadacl4*	NM_001172642	AADACL4	0	down	0	12.646
*LOC100535879*	XP_003200240		0	down	0	11.856
*LOC796876*	XP_001334272	CXorf21	0	down	0	11.856
*pou5f1*	NM_131112	POU3F3	0	down	0	11.856
*zranb3*	zranb3	ZRANB3	0	down	0	10.275
*LOC566574*	XP_001919203	GIMAP7	0	down	0	10.275

### Marginal effect of BDE47 on alternative splicing events

One advantage of deep sequencing over microarrays is its ability to detect alternative splicing, which is a common event in higher vertebrates. Therefore, the effect of BDE47 on the alternative splicing of larval transcriptome was investigated. The results are shown in Table 2. Many diseases in animals are due to abnormal alternative splicing. However, BDE47 exhibited only a marginal effect on the alternative splicing events of zebrafish larvae despite seriously affecting its transcriptome. We noticed that a similar outcome was observed in the case of benzene exposure [18]. Two genes of tropomyosin, TPM3 and TPM4, were significantly changed their alternative splicing patterns in the 500 μg/L BDE47 exposure group. Tropomyosin, which has diverse isoforms, plays a critical role in regulating the function of actin filaments; in particular, TPM3 may have a unique and vital role in embryogenesis.

**Table 2:** **Genes with significantly altered alternative splicing under 500 g/l BDE47 exposure**

**Accession ID**	**Location**	***p*** **-value**	**Splicing type**
**500 μg/l**			
*tpm3*	chr19	4.84E-03	Mutually_exclusive
*ndrg1*	chr19	6.65E-03	Altstart
*tpm3*	chr19	7.48E-03	Altstart
*ugt2a1*	chr5	9.23E-03	Altstart
*LOC560944*	chr24	9.42E-03	Cassette
*LOC100535315*	chr19	1.60E-02	Cassette
*tpm4*	chr22	2.03E-02	Altstart
*LOC560944*	chr24	3.33E-02	Mutually_exclusive
*ogt.1*	chr14	3.47E-02	Alt3
*vegfaa*	chr16	3.91E-02	Cassette
**5 μg/l**			
*plod2*	chr24	8.08E-03	Cassette
*ppap2b*	chr20	1.75E-02	Mutually_exclusive
*tnnt2e*	chr4	2.28E-02	Mutually_exclusive
*ndrg1*	chr19	4.50E-02	Altstart
*a1cf*	chr12	4.96E-02	Alt3

### Functional enrichment based on different dynamic expression patterns

**Figure 2:**
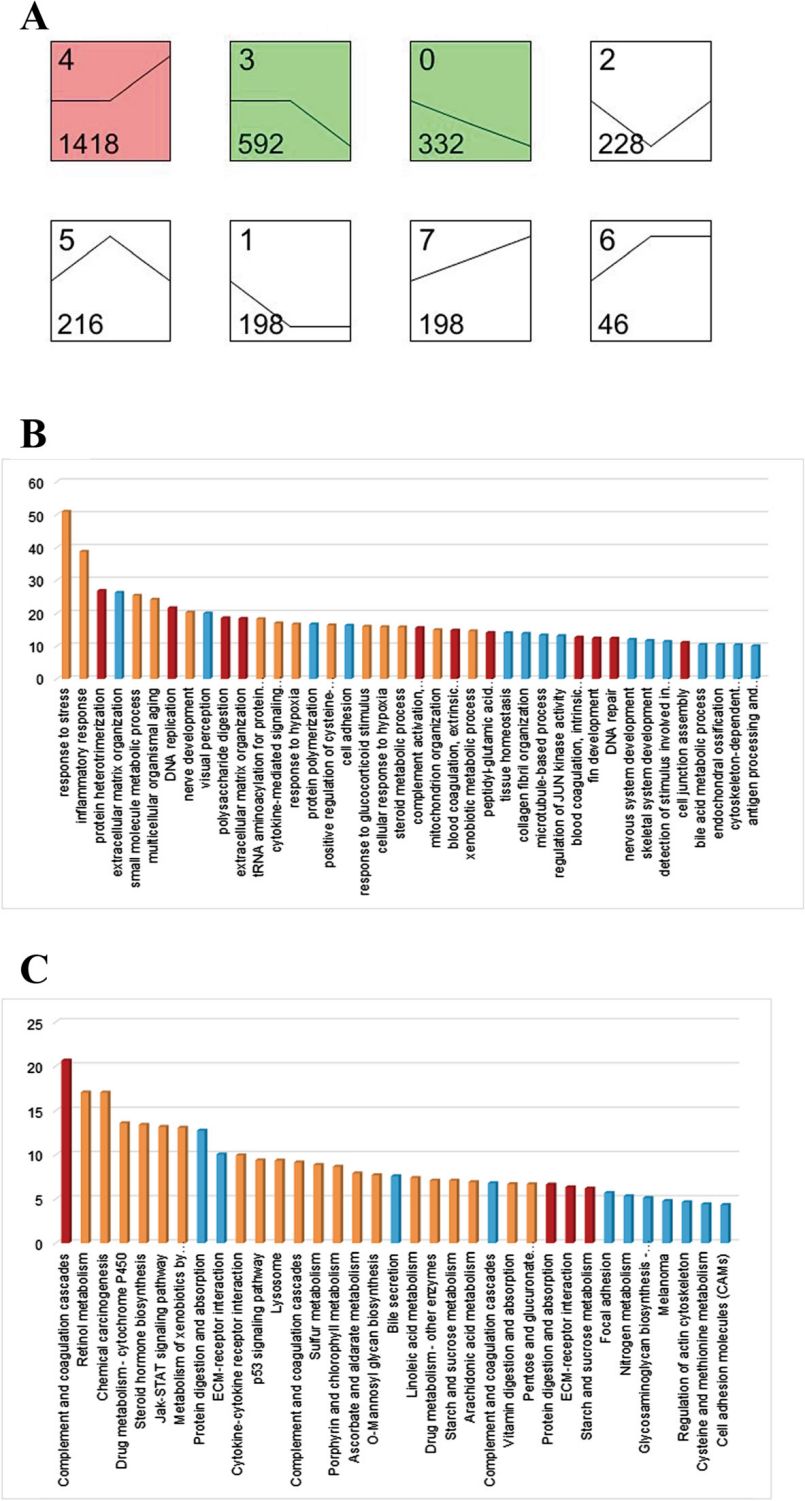
**Functional enrichment analysis based on different gene expression patterns. (A)** Expression profiles in color indicate significant ones (p < 0.05). Green, up-regulated; red, down-regulated. Profile number (up left), gene number (bottom left), and trend (line) in each profile were labelled. The significant terms were graphed using **(B)** GO annotation (p < 0.001, FDR < 0.05), and **(C)** KEGG pathway annotation (p < 0.05). Tawny block, pattern 4; teal block, pattern 3; red block, pattern 0.

All significant mRNAs were further classified into eight dynamic expression patterns under different concentrations, and the mRNA number and p-value of each pattern were calculated (Figure 2). Three patterns were significant: pattern 4 (up-regulated; only exposed to 500 μg/l BDE47), pattern 3 (down-regulated; only exposed to 500 μg/l BDE47), and pattern 0 (constantly down-regulated; exposed to both 500 and 5 μg/l BDE47). The results reaffirmed that the exposure of zebrafish larvae to high BDE47 concentration exerted a significant influence on their transcriptome, and most genes remained stable expression under low BDE47 concentration.

**Figure 3:**
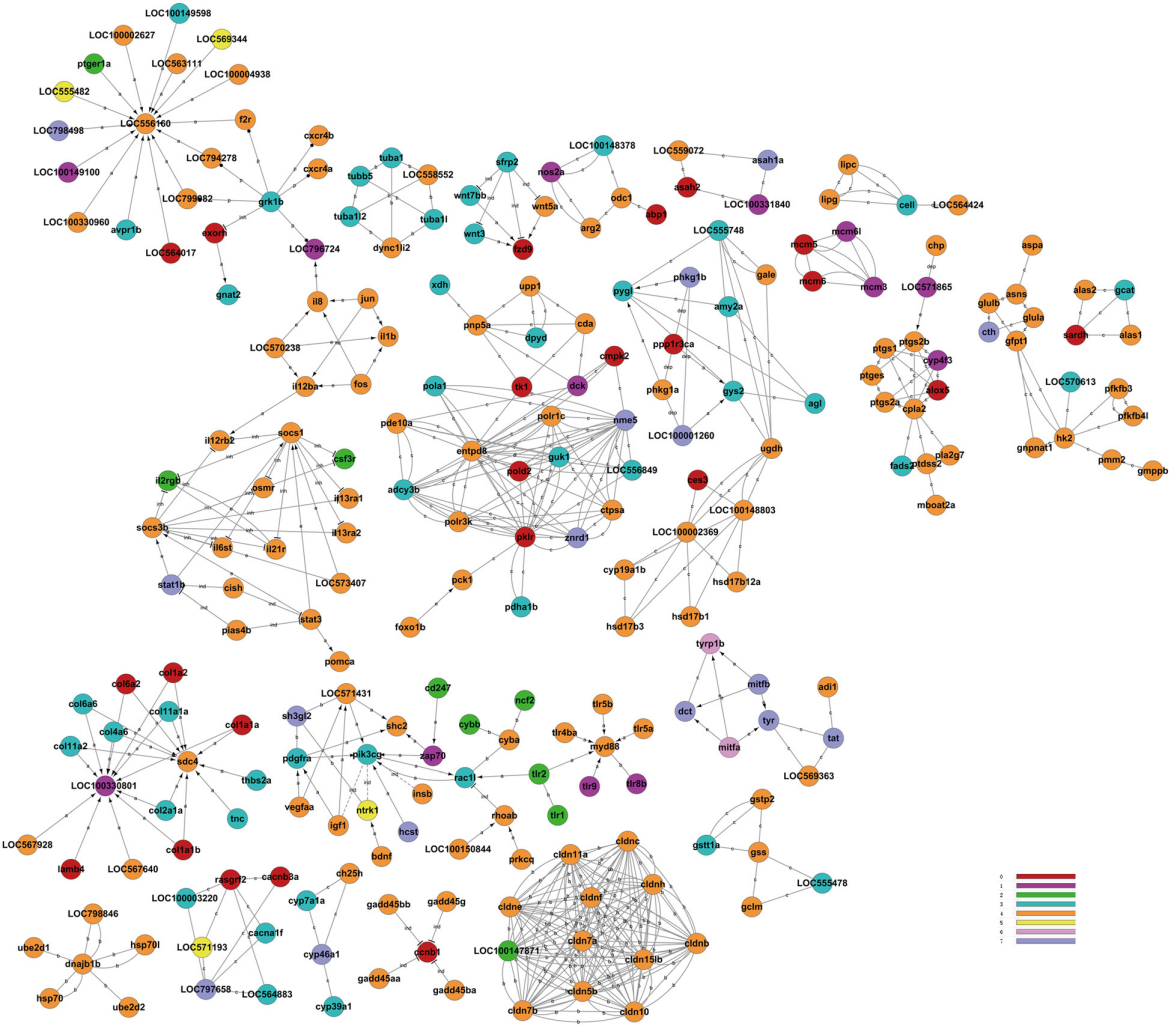
**Gene regulatory network of 6 dpf zebrafish larvae under BDE47 treatments.** The abbreviations on arrows between two nodes reflect their regulatory relationship. A, activation; b, binding/association; c, compound; dep, dephosphorylation; e, expression; ind, indirect effect; inh, inhibition; p, phosphorylation. Node colors indicate gene expression pattern. Red, pattern 0; violet, pattern 1; green, pattern 2; teal, pattern 3; tawny, pattern 4; yellow, pattern 5; lilac, pattern 6; lavender, pattern 7.

According to the expression patterns of zebrafish larvae, functional enrichment analysis was performed, and biological process ontologies were adopted in GO analysis. Because of the excessive numbers of terms obtained, the false discovery rate (FDR) was used to calibrate the p-value. The complete GO analysis results (p < 0.001, FDR < 0.05) are shown in Figure 3b. Pattern 4 represented the biological functions and processes that were significantly induced by BDE47 treatment. The affected GO terms mainly involved stress responses and metabolic processes such as: response to stress (GO:0006950); small molecule metabolic process (GO:0044281); nerve development (GO:0021675); and steroid metabolic process (GO:0008202). Patterns 3 and 0 represented the biological functions and processes that were significantly inhibited by BDE47 treatment. In pattern 3, the GO analysis results reflected BDE47 damage to the development of nerve, vision, and skeletal system in zebrafish larvae, e.g., extracellular matrix organization (GO:0030198); visual perception (GO:0007601); collagen fibril organization (GO:0030199); and skeletal system development (GO:0001501). The most enriched terms in pattern 0 included: protein heterotrimerization (GO: 0070208); extracellular matrix organization (GO:0030198); complement activation, alternative pathway (GO:0006957); and blood coagulation extrinsic pathway (GO:0007598).

KEGG pathway analysis (Figure 3c) screened out fewer terms than GO analysis, and reflected different information from that obtained by GO analysis. The significant terms in pattern 4 included: retinol metabolism (PATH:00830); chemical carcinogenesis (PATH:05204); steroid hormone biosynthesis (PATH:00140); metabolism of xenobiotics by cytochrome P450 (PATH:00980); and sulfur metabolism (PATH:00920). The affected pathway terms in patterns 3 and 0 included: protein digestion and absorption (PATH:04974); ECM-receptor interaction (PATH:04512); and glycosaminoglycan biosynthesis-chondroitin sulfate (PATH:00532). Patterns 0 and 3 had similar enrichment results. The gene regulatory network of BDE47 was constructed on the basis of the information provided by the KEGG pathway database and visualized (Figure 3).

### Validation of sequencing data

To validate the sequencing data of BDE47 treatment, the FCs of relative mRNA expression levels of several selected genes were determined by real-time quantitative PCR (RT-qPCR). Because, in the sequencing data, most of the genes significantly changed their expressions under exposure to high BDE47 concentration but showed no significant changes of their expressions under low concentration, only a RT-qPCR assay using 500 μg/L BDE47 was performed. The genes are listed in Table 3, and they include *ch25h*, *LOC799791*, *rx2*, *fetub*, *sesn2*, *opn1sw1*, *hsp70*, *cyp2k6*, *pth1a*, and *lepb*. The selected genes had significant expression changes with enough dimensions and they participated in biological functions we were concerned with, including xenobiotic metabolism (*cyp2k6*), immune response (*sesn2*, *hsp70*, *ch25h*, and *LOC799791*), endocrine regulation (*fetub*, *pth1a*, and *lepb*), eye development (*rx2*), and photosensitivity (*opn1sw1*). After RT-qPCR amplification, they were confirmed to have expression patterns similar to those observed by deep sequencing. Even though the FC values of *pth1a* and *lepb* were lower than their sequencing data, the expression changes were still dramatically significant.

**Table 3:** **Comparison of gene expression changes between deep sequencing and qRT-PCR data**

**Genes**	**RT-PCR**		**Deep sequencing**
	**FC (500)**	**SEM**	**FC (500)**
*ch25h*	4.78	1.21	4.89
*LOC799791*	1.83	0.44	2.99
*rx2*	0.43	0.07	0.18
*fetub*	0.29	0.02	0.25
*sesn2*	7.71	1.84	6.31
*opn1sw1*	0.56	0.09	0.20
*hsp70*	114.91	33.50	111.46
*cyp2k6*	5.79	0.59	10.15
*lepb*	24.97	6.24	49.47
*pth1a*	23.28	3.72	112.61

### Transcriptomic effects of DMSO vehicle

To study the possible effects of DMSO on zebrafish larvae, we compared the transcriptomes of the DMSO vehicle and blank control. Four hundred and fifty-four transcripts were significantly expressed (p < 0.05), of which 214 transcripts were up-regulated and 240 transcripts were down-regulated. Significantly changed transcripts accounted for about 2.2% of the total detected transcripts, and no abnormal phenotype was observed during larval development (data not shown). The effects of DMSO vehicle on transcriptome in our study were far weaker than those in a previous study of Turner et al. [19]. The most up-regulated gene was *crfb9* (FC = 17.19), which encoded the cytokine receptor and its human homolog displayed inhibitory properties on IL-22 effects. The expressions of 31 genes were totally inhibited, such as *cyp11a1*, *gtf3ab*, and *msh4*. Functional enrichment analysis revealed that DMSO had a major effect on inducing the immune responses of larvae. Detailed results are shown in Additional file 4: Table S4.

### Morphological and histological abnormality caused by BDE47 exposure

**Figure 4:**
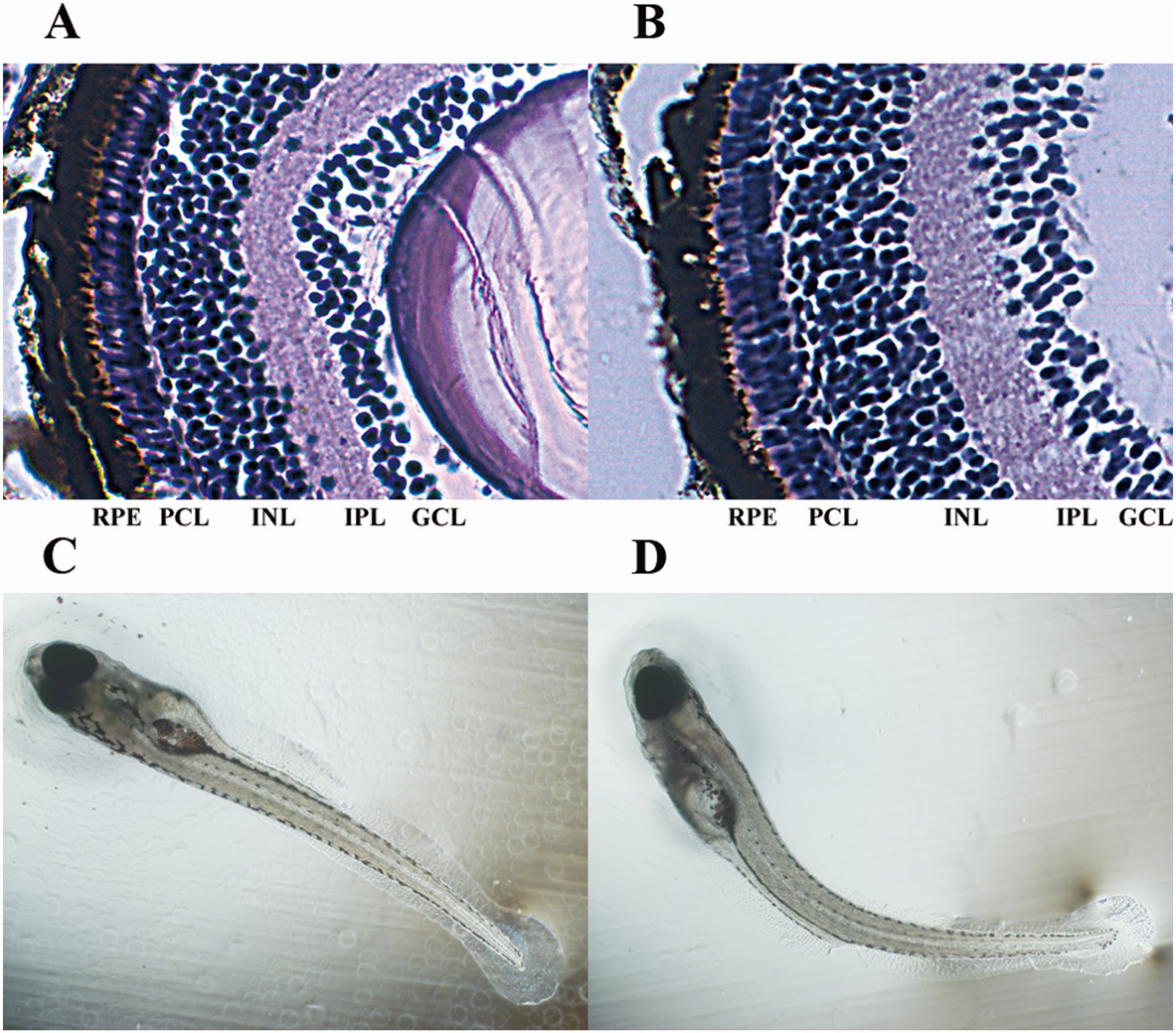
**Morphological observation of 6 dpf zebrafish larvae under BDE47 treatment and control. (A)** Normal histological patterns of larval retina (40X). **(B)** Larvae exposed to 500 μg/l BDE47 had retinal morphology distinct from the control (40X). **(C)** Normal body type of zebrafish larvae in the control group (4X). **(D)** Body curvature phenotype after 500 μg/l BDE47 exposure (4X). RPE, retinal pigment epithelium; PCL, photoreceptor cell layer; INL, inner nuclear layer; IPL, inner plexiform layer; GCL, ganglion cell layer.

To confirm the relationship between locomotor changes and disrupted larvae vision, histological sections of larval retina were prepared to investigate the effects of BDE47 on eye histology (Figure 4A-4B). In the sections exposed to 500 μg/L BDE47, a clear morphological alteration of photoreceptor cells was observed, and in the remaining sections, no significant change (data not shown) was observed compared to the control. At 6 dpf, disorderly arranged rods and cones (photoreceptors) were observed. A bipolar cell layer (BCL) was also distributed more sparsely than in the control, and the thickness of BCL increased noticeably. In general, this structural alteration of the retina closely resembled the effects caused by polychlorinated biphenyls [20], indicating that some common mechanisms probably exist among polyhalogenated aromatic hydrocarbons.

Another apparent effect of 500 μg/l BDE47 exposure on zebrafish larvae was the body curvature phenotype. Almost all larvae exhibited different degrees of curvature (88.9 ± 2.9% of malformation rate at 6 dpf); however, it was particularly evident in some individuals (Figure 4C-4D). A close examination revealed that the curvature was related to the failed inflation of the swim bladder, and we hypothesized that the curvature may result in the malformation of the swim bladder. The abnormality of the notochord and swim bladder could have implications for larval locomotion. Besides, there was no significant difference in hatching rates in both of the BDE 47 exposure groups (data not shown).

## Discussion

**Figure 5:**
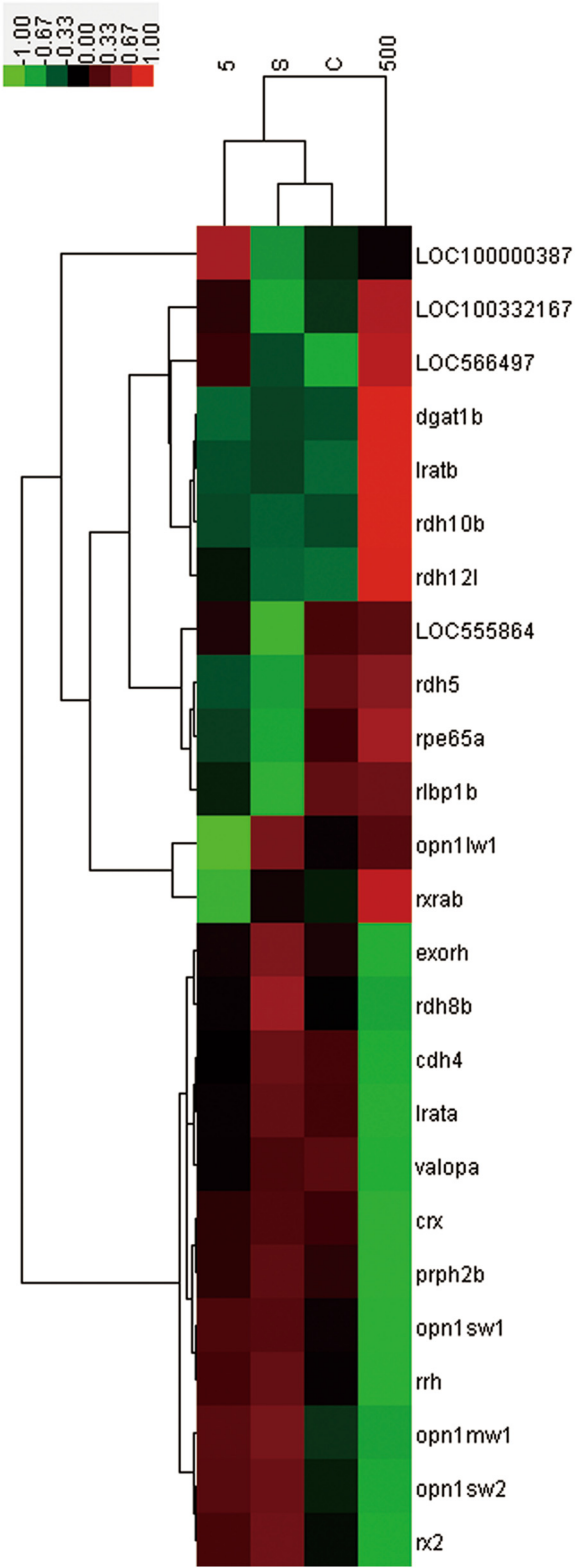
**Genes differentially transcribed related to vision formation and eye development in zebrafish larvae.** Hierarchical-clustering-analysis-based transcription levels were performed on 25 related genes showing significant differential expression (p < 0.05) in larvae. Gene tree (left) and condition tree (top) were obtained using Pearson’s uncentered distance metric calculated from all log10 transcription ratios (exposed/controls). Color scale from green to red indicate log10 ratios from −1.00 (10-fold down-regulation) to +1.00 (10-fold up-regulation). C: control; S: vehicle (0.1% DMSO); 5: 5 μg/l BDE47 treatment; 500: 500 μg/l BDE47 treatment.

According to the results of our study and previous ones, the locomotion of zebrafish was found to be less active after exposure to PBDE. Several plausible hypotheses were suggested, including disruption of the transport and absorption of the neurotransmitter acetylcholine [21], inhibition of the axonal growth of primary and secondary motor neurons [22], and deformation of the larvae [23,24]. Microscopic examination confirmed the spinal curvature of larvae under exposure to 500 μg/L BDE47. However, these above hypotheses do not explain the specific locomotor reduction during switching between light and dark conditions [11]. We believe that the most relevant cause is larval responses to light. Therefore, based on deep sequencing data, our interpretation focuses on vision. The expression levels of dozens of retina-related genes were changed by exposure to BDE47 (Figure 5). In addition, GO or KEGG terms such as visual perception and retinol metabolism had a relatively high significance in our enrichment analysis results (Figure 2).

Considering the essential roles of the conversion between retinol and retinal in the visual cycle and eye development, the up-regulation of retinol metabolism suggested that BDE47 probably affected these processes. The configurations of retinal (retinaldehyde) determine their functions during the visual cycle: in the traditional rod visual cycle, 11-cis retinal is regarded as the only chromophore. However, some evidences have revealed that in the newly discovered cone visual cycle, the retina could also use all-trans retinal directly [25,26]. The expression changes of several retinol dehydrogenase (RDH)-encoding genes were examined (Figure 5). The results revealed that expressions of 11-cis RDH genes such as *rdh5*, *rdh10b*, and *LOC555864* were up-regulated, as well as an all-trans RDH *rdh12l*. Therefore, BDE47 probably increased the photosensitive ability of zebrafish larvae and shortened their dark adaptation procedure, resulting in lack of responsiveness to changed light conditions.

The expression changes of genes in terms of visual perception indicated the possibility of abnormal eye development in larvae, which was confirmed by histological sections (Figure 5). For example, the down-regulated expressions of the cone-rod homeobox-encoding gene Crx (FC = 0.48) and the R-cadherin-encoding gene CDH4 (FC = 0.49) were found in BDE47 exposed zebarfish larvae. The deficit of the Crx function led to the failure of forming the photoreceptor outer segment (OS) and the inhibition of expressing OS-specific genes [27]. Cdh4 controls visual system development and functions of cell differentiation and axon migration of retina in cooperation with Crx [28]. Their simultaneous down-regulated expressions suggested the impaired development of the retina, especially of photoreceptor cells.

BDE47 was reported to cause abnormal dorsal curvature of the trunk and tail from 72 hpf [22], and this observation was reproduced in our experiments. We found some evidences from our sequencing results. With the exception of *hsp70.2*, *pth1a* had the most intensive expression change among all transcripts (FC = 112.61) in 500 μg/L BDE47 treated larvae. The gene encodes zebrafish parathyroid hormone (PTH), which regulates calcium and phosphorus concentrations, vitamin D metabolism, and bone cell activity [29]. Increased PTH could elevate the distribution of calcium in blood and restrain absorption of calcium into bones, as well as induce cathepsin proteins [30]. Besides those transcripts in Table 1, several transcripts encoding other cathepsin members were up-regulated (FC < 10) their expressions in the complete differential expression analysis results. Because of the key function of cathepsins in bone resorption, these changes indicated that the formation of larval spinal curvature was probably caused by the restricted absorption of calcium in the bone via the cooperation of PTH and cathepsins. Our functional enrichment analysis (Figure 3b) also reflected the adverse impacts of BDE47 on the larval skeletogeny process.

PBDEs are known for their ability to interrupt the production, transport, and metabolism of T3 and T4. However, our studies showed no evidence that expressions of TH genes were affected by BDE47, including genes encoding TH receptors, transthyretin, and T3 deiodinases. Different effects of BDE47 were seen in adults and larvae. During development, the zebrafish thyroid forms the first follicle from around 60 hpf [31], and all the observed TH changes occurred in zebrafish larvae older than that stage. These observations pose some interesting questions: when and why did BDE47 begin to disrupt zebrafish THs? What role did BDE47 play on the total thyroid system of the zebrafish larvae? The only affected hormone in our experiment was calcitonin, which is also secreted from the thyroid; the expression of its gene *calca* was moderately up-regulated (FC = 2.37). Calcitonin can both respond to PTH, and antagonize the biological effects of PTH [32]. Therefore, in 6 dpf larvae, the primary influence of BDE47 on the thyroid was also relevant to the disturbance of bone formation.

## Conclusions

In general, exposure to BDE47 resulted in the body curvature phenotype and abnormal locomotion. Distinct from the reported explanation focused on neurological factors, our transcriptomic analysis revealed that the phenotypes resulted from the exposure of BDE47 were due to disrupt the vision and bone development in zebrafish larvae. Light was a critical factor regulating fish behavior. Hence, altered photosensitivity and locomotion were anticipated to have significant consequences on the prey, avoidance, and survival of zebrafish larvae. This study proposes the larval retina as a potential target organ of BDE47 exposure. Although the toxicological effects identified in this study resulted from high levels of exposure, it is likely that our findings will be helpful for understanding the underlying mechanisms and developmental consequences of the toxicity of BDE47 and other PBDE congeners in environmental situations featuring lower concentrations, but longer terms and more complex pollutant mixtures, and therefore merit further investigation.

## Methods

### Zebrafish

Wild-type Tuebingen zebrafish (Danio rerio) were placed in a recirculating filtration system using water treated by reverse osmosis (ESEN, China). Zebrafish were fed with live brine shrimp twice a day, and aquarium flake food (OSI, Hayward, CA) once a day during the spawning period. The system was maintained at 28.5°C with a 14-h light/10-h dark cycle. The main water quality parameters, such as pH, temperature, conductivity, and ammonia-N (NH_3_-N) were monitored weekly. Adult fish aged from 4 to 6 months were chosen for spawning. The embryos were collected within the first hour of lighting and rinsed with the water from filtration system. All related protocols were approved by the Animal Ethics Committee of Tongji University.

### Toxicological exposure and morphological examination

BDE47 (purity > 99%, Accustandard, New Haven, CT) was dissolved in dimethyl sulfoxide (DMSO) vehicle (purity > 98%, Amresco, Solon, OH). The final concentrations of BDE47 were 0, 5, and 500 μg/L of medium with 0.1% vehicle, respectively. A sample without a vehicle was also set as a negative control for zebrafish development. To maintain consistency with our previous behavioral assay [11], exposure in an incubator started from 3 to 4 hours post-fertilization (hpf) at 28.5°C. Exposure solutions were replaced 50% daily to minimize the changes of BDE47 concentrations. In each treatment, approximately 60 larvae exposed until 6 dpf were used for deep sequencing and RT-qPCR. Deep sequencing was performed once with the total RNAs isolated from the 6 dpf larvae exposed with BDE47 and DMSO started from 3 to 4 hpf whereas RT-qPCR assays were performed three times with the total RNA isolated from three different batches of the 6 dpf larvae exposed with BDE47 and DMSO, respectively. Approximately 30 embryos/larvae were used in triplicate for continuous morphological monitoring using an Olympus IX71 reversed microscope (Olympus, Japan) with a DP72 digital camera.

### Total RNA isolation and sequencing experiments

The total RNA was extracted using TRIzol reagent (Invitrogen, Carlsbad, CA) and purified by the RNeasy Mini Kit (Qiagen, Germany) according to the manufacturer’s instructions. The quality of the total RNA was examined using an Agilent 2200 TapeStation (Agilent Technologies, Santa Clara, CA) and then RNA library and sequencing was performed by the Illumina HiSeq 2000 platform (Illumina, San Diego, CA). In detail, the total RNA of zebrafish larvae was purified using Sera-Mag Oligo (dT) beads (Thermo Scientific, Indianapolis, IN) after DNase I treatment. The mRNA was fragmented by heating and then treating with sodium acetate. The cleaved RNA fragments were transcribed into first-strand cDNA using reverse transcriptase, followed by second-strand cDNA synthesis employing DNA polymerase and RNase H and cDNA purification using the QIAquick Gel Extraction kit (Qiagen, Valencia, CA). The double-stranded cDNA was further subjected to end repair employing T4 DNA polymerase, the Klenow fragment, and T4 polynucleotide kinase followed by a poly(A)-tailing procedure using Klenow exo-polymerase and ligation with an adapter using T4 DNA ligase. Adapter-ligated fragments were separated by agarose gel electrophoresis, and the desired range of cDNA fragments (200 ± 25 bp) was excised from the gel and enriched by PCR to construct the final cDNA library. After validation with Agilent 2200, the cDNA library was pair-end sequenced on a flow cell using Illumina HiSeq 2000.

### Differential expression analysis and gene enrichment analysis

After obtaining sequences from the raw sequencing data, clean reads were mapped to the reference zebrafish genome (Zv9 assembly) and *H. sapiens* (hg19 assembly) using Basic Local Alignment Search Tool (BLAST) to increase the subsequent annotation efficiency. Mapped data were then normalized using the trimmed mean of M-values (TMM) normalization method. The statistical analysis of differential expression was performed by the DEGseq package in the R language [33], and “significant” was defined as significance level p < 0.05 and a fold change (FC) of 2. Gene functions were defined and annotated using gene ontology (GO) terminology (http://www.geneontology.org) and Kyoto encyclopedia of genes and genomes (KEGG) pathway (http://www.genome.jp/kegg/pathway.html). To statistically identify differential gene sets between the control and treatments, the Fisher exact test was used for GO analysis (*p* < 0.001) and KEGG pathway analysis (*p* < 0.05). Differential alternative splicing events were calculated by employing the methodology of Zhou et al. [34], and the significance level was set as *p* < 0.05. The gene regulatory network was constructed on the basis of the information obtained from the KEGG pathway database and visualized by Cytoscape, which is a common open source software package.

### Real-time quantitative PCR

After RNA quality control by gel electrophoresis and biophotometer (Eppendorf, Germany), the total RNA was reverse-transcribed into cDNA with Superscript III reverse transcriptase (Invitrogen), random hexamers, and oligo(dT) primers. SYBR Green I qPCR was performed using a 7500 Fast Real-Time PCR system (Applied Biosystems, Foster City, CA). The PCR amplification mixture contained 0.2 μL platinum Taq polymerase, 2 μL PCR buffer (10×), 0.5 μL forward primers, 0.5 μL reverse primers, 1 μL SYBR Green dye (20×), and 1 μL reverse transcription products to make a final volume of 20 μL. PCR conditions were 40 cycles of 95°C for 10 s, 60°C for 30 s, and 70°C for 30 s, and melting curves were generated after the last cycle. The threshold cycle values for selected genes and the housekeeping RPL13a gene were used to acquire the amount of RNA equivalents after the comparison with other alternatives like β-actin and 16S RNA. Primers for RT-qPCR were designed using the Primer Express software (Applied Biosystems, Foster City, CA); they are shown in Additional file 5: Table S5.

### Histological observation

First, fresh zebrafish larvae samples were fixed overnight in formalin/acetic acid/70%alcohol (18:1:1) at 4°C. Next, they were dehydrated using an alcohol dilution series (70%, 80%, 90%, 95%, followed by 100%). Finally, the samples were embedded into 100% paraffin. Serial sections were cut to a thickness of 3 μm and stained with hematoxylin-eosin staining. The slides were examined using a Motic BA310 microscope (Motic, Germany) with a Moticam Pro digital camera.

### Availability of supporting data

The RNA sequencing data have been deposited in Gene Expression Omnibus with accession number GSE59968, which are available at http://www.ncbi.nlm.nih.gov/geo/query/acc.cgi?acc=GSE59968.

## Additional files

Additional file 1: Table S1. The most abundant transcripts in four groups. Additional file 2: Table S2. The differentially expressed genes of zebrafish larvae treated by 500 μg/L BDE47 (*p* < 0.05). Additional file 3: Table S3. The differentially expressed genes of zebrafish larvae treated by 5 μg/L BDE47 (*p* < 0.05). Additional file 4: Table S4. The differentially expressed genes of zebrafish larvae between DMSO solvent control and negative control (*p* < 0.05). Additional file 5: Table S5. Primers used for quantitative PCR amplification.
